# *Chrna7* deficient mice manifest no consistent neuropsychiatric and behavioral phenotypes

**DOI:** 10.1038/srep39941

**Published:** 2017-01-03

**Authors:** Jiani Yin, Wu Chen, Hongxing Yang, Mingshan Xue, Christian P. Schaaf

**Affiliations:** 1 Department of Molecular and Human Genetics, Baylor College of Medicine, Houston, TX 77030, USA; 2 Jan and Dan Duncan Neurological Research Institute at Texas Children’s Hospital, Houston, TX 77030, USA; 3 Department of Neuroscience, Baylor College of Medicine, Houston, TX 77030, USA; 4 The Cain Foundation Laboratories, Jan and Dan Duncan Neurological Research Institute at Texas Children’s Hospital, Houston, TX 77030, USA; 5 Shanghai Chenshan Plant Science Research Center, Shanghai Chenshan Botanical Garden, Chinese Academy of Sciences, Shanghai, 201602, China

## Abstract

The alpha7 nicotinic acetylcholine receptor, encoded by the *CHRNA7* gene, has been implicated in various psychiatric and behavioral disorders, including schizophrenia, bipolar disorder, epilepsy, autism, Alzheimer’s disease, and Parkinson’s disease, and is considered a potential target for therapeutic intervention. 15q13.3 microdeletion syndrome is a rare genetic disorder, caused by submicroscopic deletions on chromosome 15q. *CHRNA7* is the only gene in this locus that has been deleted entirely in cases involving the smallest microdeletions. Affected individuals manifest variable neurological and behavioral phenotypes, which commonly include developmental delay/intellectual disability, epilepsy, and autism spectrum disorder. Subsets of patients have short attention spans, aggressive behaviors, mood disorders, or schizophrenia. Previous behavioral studies suggested that *Chrna7* deficient mice had attention deficits, but were normal in baseline behavioral responses, learning, memory, and sensorimotor gating. Given a growing interest in *CHRNA7*-related diseases and a better appreciation of its associated human phenotypes, an in-depth behavioral characterization of the *Chrna7* deficient mouse model appeared prudent. This study was designed to investigate whether *Chrna7* deficient mice manifest phenotypes related to those seen in human individuals, using an array of 12 behavioral assessments and electroencephalogram (EEG) recordings on freely-moving mice. Examined phenotypes included social interaction, compulsive behaviors, aggression, hyperactivity, anxiety, depression, and somatosensory gating. Our data suggests that mouse behavior and EEG recordings are not sensitive to decreased *Chrna7* copy number.

The alpha7 nicotinic acetylcholine receptor (α7 nAChR) is widely expressed in the peripheral and central nervous systems, immune system, and other peripheral tissues^1^,^2^,^3^,^4^. It has been implicated in various psychiatric and behavioral disorders, including schizophrenia, bipolar disorder, epilepsy, autism, Alzheimer’s disease, and Parkinson’s disease^5^,^6^,^7^,^8^,^9^,^10^,^11^. In addition, individuals with genomic copy number changes involving the *CHRNA7* gene have been described^12^,^13^,^14^,^15^. Those with 15q13.3 microdeletion syndrome, caused by heterozygous deletions involving the *CHRNA7* gene, manifest variable neurological and behavioral symptoms, such as cognitive impairments, epilepsy, deficits in social interaction, decreased attention spans, and aggressive behaviors. Subsets of patients carry psychiatric diagnoses, such as autism spectrum disorder, mood disorders, and schizophrenia. It is important to understand the significance of alpha7 nAChR in the context of normal brain function, behavior, and disease.

Animal models have been proposed as powerful tools in assessing the causal relationship between genetic components and disease phenotypes. The α7 nAChR has been knocked out in mice by deleting exons 8–10, which encode the bulk of its transmembrane domains^16^. Initial physiological and behavioral characterizations of these mice found that they were grossly normal, with no deficits in sensorimotor reflexes, anxiety levels, locomotor activity, motor function, learning and memory, or auditory sensory gating^17^. Yet subsequent studies reported that *Chrna7* knockout mice did have impaired working memory, attention, and visual acuity^18^,^19^,^20^,^21^,^22^. To our knowledge, no existing report addresses the effect of *Chrna7* deficiency on autism-like features, depression-like behaviors, aggression, or spontaneous electroencephalogram (EEG) activity in animal models. Furthermore, reports on anxiety levels and auditory sensory gating of *Chrna7* deficient mice have been largely inconsistent^17^,^21^,^23^.

To evaluate the putative role of *Chrna7* in a variety of neuropsychiatric and behavioral phenotypes, we compared *Chrna7* deficient mice of each sex with wildtype littermates, using a battery of 12 behavioral assays and EEG/EMG recordings. We show that *Chrna7* deficient mice demonstrate subtle phenotypes, if any, in a variety of behavioral assays, suggesting that *Chrna7* is not essential for social interaction, or control over emotions and behavior in mice.

## Methods

### Animals

Mice were maintained on a 14 hr light/10 hr dark cycle, with access to regular mouse chow and water *ad libitum*. For behavioral assessments, homozygous mutants (C57BL/6 J background, B6.129S7-*Chrna7*^*tm1Bay*^/J, number 003232) were purchased from The Jackson Laboratory and then bred to wildtype mice (C57BL/6 J) to obtain heterozygous mice for breeding pairs. All of the *Chrna7* homozygous (KO) and heterozygous (HET) mutant mice, and wildtype (WT) mice used in our experiments were derived from HET breeding pairs. Mice were randomly assigned and group-housed, with two to five animals per cage, immediately after weaning. Cohort one, consisting of 14 WT (♂), 13 HET (♂), 15 KO (♂), 12 WT (♀), 15 HET (♀), 12 KO (♀), went through a series of assays, including: elevated plus maze, open field, self-grooming, holeboard exploration, marble burying, three-chamber test, partition test, reciprocal social interaction, and prepulse inhibition, all of which started at 10 weeks of age. Cohort two, consisting of 15 WT (♂), 21 HET (♂), 13 KO (♂), 13 WT (♀), 19 HET (♀), 18 KO (♀), were tested in tail suspension, forced swimming, and tube tests, which began at 11.5 weeks of age. Experiments were performed during the light cycle, and in between each test, mice were given inter-test intervals of 2–3 days. All behavioral tests were performed at 700–750 lux illumination and background white noise at approximately 60 dB, with the exception of the partition test, in which there was no background white noise.

For EEG/EMG recording, *Chrna7* deficient mice were backcrossed locally to sighted FVB mice purchased from The Jackson Laboratory (FVB.129P2-*Pde6b*^+^ *Tyr*^*c-ch*^/AntJ, Jackson Laboratory stock number 004828), which provided a phenotype-sensitizing background based on the experience of Dr. Jianrong Tang from the Baylor College of Medicine Intellectual and Developmental Disabilities Research Center Neuroconnectivity Core. To speed up the backcrossing process, we sent snap frozen tails of mice from each generation to The Jackson Laboratory and seleted the breeder based on scores from the speed congenic service (Jackson Laboratory). After 4 generations, we obtained mice 94–97% congenic to FVB background. The WT and KO mice used in experiments were obtained from heterozygote breedings of this generation.

For all assays, the experimenter remained blind to the genotypes. All research and animal care procedures were approved by the Baylor College of Medicine Animal Care and Use Committee, and were performed in accordance with the relevant guidelines and regulations.

### Genotyping

The last 1–2 mm of mouse tails were cut into a 1.5 ml Eppendoff tube containing 135 μl 50 mM NaOH and were incubated overnight on a 55 °C shaker. DNAs were extracted by adding 15 μl Tris-HCL pH 6.8 and centrifuging at 20,000 × g for 1 min.

1 μl of DNA was used for each PCR reaction using Platinum taq DNA polymerase (Thermo Fisher Scientific) and a combination of forward primer with one of two reverse primers: forward primer 5′- TTCCTGGTCCTGCTGTGTTA, reverse primer for wildtype 5′- ATCAGATGTTGCTGGCATGA, reverse primer for mutant 5′- TAGCCGAATAGCCTCTCCAC.

### Elevated plus maze

Anxiety was assessed using the elevated plus maze, as described previously^24^, with a few modifications. Mice at 10–11 weeks of age were put at the cross area of the maze in white, facing the open arm. The maze was elevated 50 cm above the floor. Activity data was collected over a 10 min period, using the Fusion software (AccuScan Instruments, Columbus, OH, USA) version 4.75.

### Open field assay

Locomotor activity and anxiety level were assessed at 11.5 weeks of age, using the open field assay, as described previously^25^, with the following modifications: activity in a clear acrylic (40 cm × 40 cm × 30 cm) open field arena was recorded over a 30 min period, using the Fusion software version 3.7.

### Self-grooming

The self-grooming test was used to evaluate compulsive grooming behaviors, as described previously^26^. Each mouse was placed individually into a standard mouse cage with a thin layer of bedding, habituated for at least 30 min, and was then videotaped for 10 min. Time spent on spontaneous grooming of any part of its face, body, limbs, or tail was quantified and subsequently analyzed.

### Holeboard exploration

The holeboard exploration test was used to evaluate repetitive nose-poke behavior. Mice at 12.5 weeks of age were placed into a clear acrylic (40 cm × 40 cm × 30 cm) chamber with a black 16-hole floorboard. Holeboard exploration data was collected, using the Fusion software version 7.7. The number of total and sequential nose-pokes in a 10 min period was quantified.

### Marble burying

The marble burying test was used to evaluate repetitive digging behavior at 13 weeks of age, as described previously^27^, with the following modifications: corncob bedding was used, and a 30 min exploration period was allowed before mice were carefully removed from the cage and the number of marbles that were more than 50% buried were counted.

### Three-chamber test

Sociability was assessed at 15 weeks of age, using the three-chamber test, as described previously^28^, with a few modifications. After a 10 min habituation period, a sex- and age-matched C57BL/6 J mouse was placed under one wire cup, and a lego object of similar size and color was placed under the wire cup in the opposite compartment. Test mice were then allowed to explore freely for another 10 min. Data of time spent in each compartment, and the amount of time in close contact with each wire cup in the two phases were determined, using ANY-Maze version 4.75 and manual scoring.

### Partition test

Interest in social novelty was assessed, using the partition test, as described previously^29^, with a few modifications. At 16 weeks of age, each test mouse was housed overnight with an age- and sex-matched C57BL/6 J partner mouse in the two separate compartments of a partition cage. The next day, activity at the partition board was measured, first with the familiar overnight partner, followed by an unfamiliar partner, and then back to the original familiar partner, for 5 min each. This was manually scored using a Psion Handheld Computer and Observer XT (Noldus Information Technology, Netherlands).

### Reciprocal social interaction

After the partition test, mice continued to be housed in the partitioned cages. The next day, the partition was removed, and the filter-top lid was replaced by a clear perforated board. Direct interactions between the test and partner mice were videotaped from above for 10 min. Non-social, active, and passive social behaviors were scored later, as described previously^30^. Non-social behaviors include walking, sitting, sleeping, grooming, digging, etc. Passive social behaviors include escaping, freezing, and displaying defensive behaviors when approached by a partner mouse. Active social behaviors include face/body sniffing, anogenital sniffing, non-aggressive contact, and aggressive contact. Durations of each behavior were manually scored using Psion Observer XT.

### Prepulse inhibition

The prepulse inhibition (PPI) test was used to evaluate schizophrenia-associated behavior at 16.5 weeks of age, as described previously^25^. But instead of five prepulse sounds of different intensity, only three (74, 78, 82 dB) were presented, using the SR-LAB Startle Response System (San Diego Instruments, San Diego, CA, USA) version 5. Percent prepulse inhibition of the startle response was calculated for each acoustic prepulse intensity as 100 − [(startle response on the prepulse plus startle stimulus/startle response alone) × 100].

### Tail suspension test

Depression-related behavior was assessed at 11.5 weeks of age, using the tail suspension test, as previously described^25^. Mouse tails were stuck to a shelf-overhang, which was elevated 30 cm from the table beneath it. The time spent immobile was automatically determined by the ANY-Maze Video Tracking System version 4.75 (Stoelting Co., Wood Dale, IL, USA).

### Forced swimming test

Depression-related behavior was assessed using the forced swimming test. Mice at 12 weeks of age were placed into a 22 cm diameter circular tank with 17 cm deep water at room temperature (25 °C) for 6 min. Immobility time was defined as the duration in which the percentage of immobility was greater than 88% during any 500 msec period. This was automatically determined using the ANY-Maze Video Tracking System version 4.75 (Stoelting Co., Wood Dale, IL, USA).

### Tube test

Aggression and social dominance were assessed using the tube test. Test mice at 12.5 weeks of age and sex- and age-matched C57BL/6 J wildtype partner mice were placed headfirst, at opposite ends of a clear plastic tube (3.1 cm inner diameter for males, 2.6 cm inner diameter for females, 30.5 cm in length) and released simultaneously. The match ended when one mouse completely retreated from the tube. The mouse remaining in the tube was designated as the winner (score = 1), and the one that retreated from the tube was designated as the loser (score = 0). Each test and partner mouse were subjected to three matches, each time with a different opponent.

### Behavioral data analysis and statistics

Statistical analysis of sex and genotype effects on all behavioral studies were performed using Two-Way ANOVA analysis of variance (ANOVA), linear mixed mode, or the Wald Chi-square test, where appropriate (R package). We used aligned rank transform (ART) as a procedure to preprocess data when it did not meet the assumptions of ANOVA. This occurred when the residuals did not follow a normal distribution. The Aligned Rank Transform (ART) method is suitable for nonparametric factorial data analysis, including the evaluations of the interaction effects^31^,^32^,^33^. The preprocessing step “aligns” data for each factor and interaction before applying averaged ranks. After this process, common ANOVA procedures can be used. We recruited methods implemented in the R package ARTool for ART (R package version 0.10.2). If an ANOVA was significant, a Tukey’s HSD *post hoc* test or *post hoc* interaction analysis (phia) was performed for between-group comparisons or interaction analysis. P values of <0.05 were considered to be statistically significant. All data was presented as mean ± SEM (GraphPad Prism 6.0e, La Jolla, CA, USA).

### Surgery and EEG recordings

Video-EEG and EMG were acquired from 1 WT (♂), 2 KO (♂), 4 WT (♀), 5 KO (♀) animals at 3 months of age by the Neuroconnectivity Core at Baylor College of Medicine. The methods were modified from previous publications^35^. Adult mice at 10 weeks were anesthetized with 1–2% isoflurane. Under aseptic conditions, each mouse was surgically implanted with the cortical EEG recording electrodes (Teflon-coated silver wire, 127 μm diameter) in the subdural space of the left frontal cortex and the right parietal cortex, respectively, with the reference electrode positioned in the occipital region of the skull. The third recording electrode (Teflon-coated tungsten wire, 50 μm diameter) was aimed at the dentate gyrus (P2.0R1.8H1.8), with the reference electrode at the corpus callosum. In addition, the fourth recording electrode (silver wire) was inserted into the neck muscles, to monitor the electromyogram (EMG) as an indicator of animal activity level. All electrode wires were attached to a miniature connector (Harwin Connector) and secured on the skull by dental cement. After 2 weeks of post-surgical recovery, simultaneous EEG activity, EMG activity (filtered between 0.1 Hz and 1 kHz, sampled at 2 kHz), and behavior were recorded in freely moving mice for 2 hours per day over 4 days.

### EEG data analysis

Video-EEG and EMG were visually inspected to identify electrographic seizures. For power spectral density (PSD) analysis, EEG signals were divided into segments (10 minutes per segment) and any segment containing artifacts was excluded. The results were obtained from 406 ± 30 and 317 ± 22 (mean ± SEM) minutes of EEG data for WT and KO mice, respectively. Each segment of the EEG data was first detrended by computing the least-squares fit of a line, and subtracting the line from the EEG data. EEG signals were then filtered by a high-pass filter at 0.5 Hz and a notch filter at 60 Hz. The PSD was calculated using Fast Fourier Transform (FFT) from each segment in the frequency domain, and was then averaged across all segments for each mouse. The PSD was then averaged within each of the following frequency bands: 1–4 Hz, 4.5–8.5 Hz, 9–14 Hz, 14.5–30 Hz, 30.5–70 Hz, 70.5–250 Hz, and 250.5–500 Hz, which correspond to the delta, theta, sigma, beta, gamma, ripple, and fast ripple ranges, respectively. The electrode in the parietal cortex of 1 WT mice was defective, hence the data of the parietal cortex were from 4 KO mice. All computations were performed in MATLAB R2013b and Repeated Measures Two-Way ANOVA with Sidak’s multiple comparison tests were used for statistical analysis in Graphpad Prism 6.

## Results

**Figure 1:**
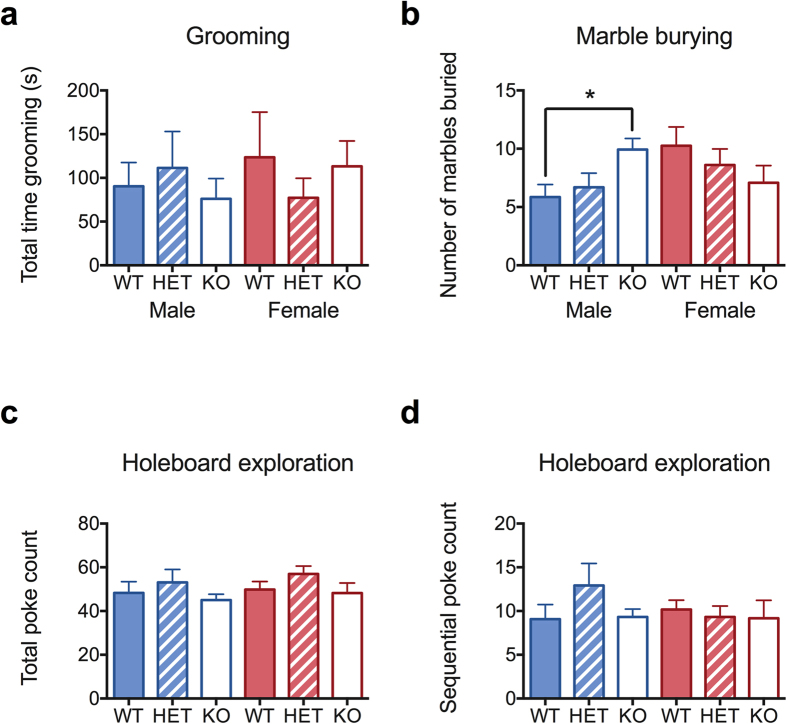
Repetitive behaviors in *Chrna7* deficient and wildtype mice. *N* = 12–15. (**a**) Self-grooming, assessed over a 10 min period. (**b**) Marble burying. Number of marbles buried during a 30 min period. (**c**,**d**) Holeboard assay. Number of total nose pokes and total sequential nose pokes in each hole. Each point represents the mean ±  SEM (*P < 0.05).

In an attempt to address whether *CHRNA7* has an effect on repetitive behaviors and restricted interests, two of the core phenotypes of individuals with autism spectrum disorder, we tested homozygous and heterozygous *Chrna7* deficient mice and their wildtype littermates in self-grooming, holeboard nose-poking, and marble burying tests. As can be seen in Fig. 1a, there was no significant difference in self-grooming time among gentoypes (Two-WAY ANOVA, *F* [2,75] = 0.11, *P* = 0.90), or between males and females (*F* [1,75] = 0.17, *P* = 0.68). The genotype x sex interaction was not significant (*F* [2,75] = 0.76, *P* = 0.47). In the marble burying test (Fig. 1b), there was a significant effect from genotype x sex interaction, which came from the difference between KO and WT between the two sexes (Aligned rank transformation followed by Two-Way ANOVA, genotype effect *F* [2,75] = 0.26, *P* = 0.77; sex effect *F* [1,75] = 0.10, *P* = 0.33; sex x genotype *F* [2,75] = 4.32, *P = 0.02, post-hoc interaction analysis revealed difference between male: (KO-WT) and female: (KO-WT) *F* [1,11] = 8.48, multiple-test corrected *P = 0.01). In the holeboard nose-poking assay, *Chrna7* deficient mice were not significantly different from wildtype mice (Fig. 1c,d, Two-Way ANOVA on total number of nose-pokes, genotype effect *F* [2,75] = 2.01, *P* = 0.14; sex effect *F* [1,75] = 0.66, *P* = 0.41; genotype x sex interaction *F* [2,75] = 0.04, *P* = 0.96; Aligned rank transformation followed by Two-Way ANOVA on number of sequential nose-pokes, genotype effect *F* [2,75] = 0.72, *P* = 0.82; sex effect *F* [1,75] = 0.53, *P* = 0.45; genotype x sex interaction *F* [2,75] = 2.41, *P* = 0.10). Taken together, results of the three repetitive behavior assays suggest that *Chrna7* deficiency does not cause impulsive behaviors in general, even though we reach significance with a genotype by sex effect in the marble burying test.

**Figure 2:**
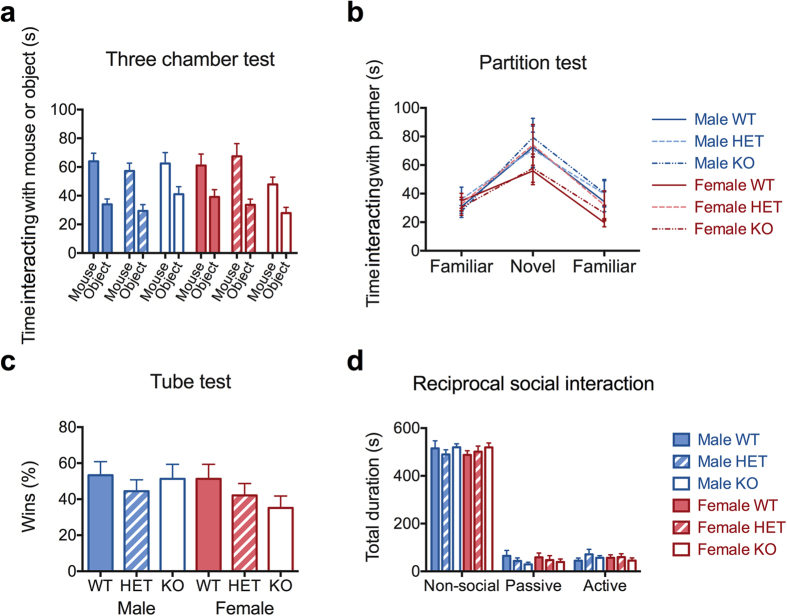
Social behaviors in *Chrna7* deficient and wildtype mice. (**a**) Time in close contact with the mouse or the object in three-chamber test, as an indication of sociability. *N* = 12–15. (**b**) Time in close contact with the partition board with familiar or novel partner mice on the other side in partition test, as an indication of appreciation of social novelty. *N* = 12–15. (**c**) Percent wins in the tube test, as an indication of social dominance or aggression. *N* = 13–21. (**d**) Time spent on non-social behaviors, passive social and active social behaviors in the reciprocal social interaction test. *N* = 12–15. Each point represents the mean  ±  SEM.

Impairment in social interaction is another core phenotype of individuals with autism spectrum disorder. To address whether loss of *CHRNA7* has an effect on social behaviors, we tested homozygous and heterozygous *Chrna7* deficient mice and their wildtype littermates in four different assays, each having a slightly different purpose. Figure 2a shows that *Chrna7* deficient mice maintain preference of social versus nonsocial objects (Linear mixed mode followed by evaluation of significance by ANOVA, genotype effect *F* [2,75] = 0.44, *P* = 0.64; sex effect *F* [1,75] = 0.24, *P* = 0.63; object effect *F* [1,75] = 58.18, *P = 6.09e-11; genotype x sex interaction *F* [2,75] = 3.31, *P = 0.04; genotype x object effect *F* [2,75] = 0.77, *P* = 0.47; sex x object effect *F* [2,75] = 0.003, *P* = 0.96; genotype x sex x object effect *F* [2,75] = 0.36, *P* = 0.70). There was also no difference in appreciation of social novelty, as can be seen in Fig. 2b (Linear mixed mode followed by evaluation of significance by ANOVA, genotype effect *F* [2,75] = 0.42, *P* = 0.66; sex effect *F* [1,75] = 1.60, *P* = 0.21; genotype x sex *F* [2,75] = 0.20, *P* = 0.82; partner or time effect *F* [2,160] = 46.47, *P < 0.0001). Given that a number of male 15q13.3 microdeletion patients manifest aggressive behaviors, we also tested the *Chrna7* deficient mice for social dominance and aggression, using the tube test. We found that *Chrna7* null mice did not manifest increased levels of aggression (Fig. 2c, Logistic regression followed by Wald Chi-square test, genotype effect X^2^(2) = 2.4, *P* = 0.30; sex effect X^2^(1) = 0.07, *P* = 0.80; genotype x sex X^2^(2) = 1.2, *P* = 0.54). Lastly, we wanted to expand our behavioral observations by using a less structured assessment, so we allowed the subject mice to interact freely with a partner mouse, and videotaped their behaviors for a period of 10 min. We then determined the time that the subject mice spent on a range of non-social and social behaviors (Fig. 2d, and data not shown). Results from this reciprocal social interaction test did not display abnormalities in social behaviors of the mice assessed (Three-Way ANOVA, genotype effect *F* [2,225] = 0.05, *P* = 0.95; sex effect *F* [1,225] = 0.06, *P* = 0.80; all *P* values for effect of between-factor interactions were above 0.1). In conclusion, we found that *Chrna7* deficient mice display grossly normal social interactions.

**Figure 3:**
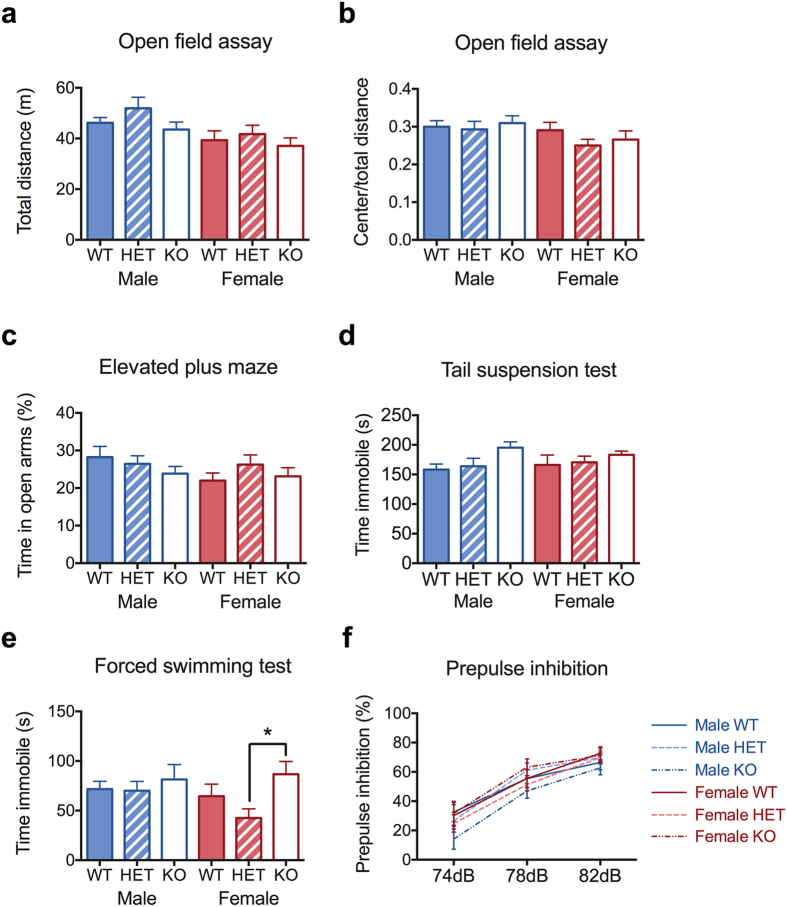
Phenotypes related to human neuropsychiatric disease, assessed in *Chrna7* deficient and wildtype mice. (**a**,**b**) Total distance and center-to-total distance ratio in the open field assay, as an indication of locomotor activity and anxiety level, respectively. *N* = 12–15. (**c**) Percent time in the open arms in the elevated plus maze, as an indication of anxiety. *N* = 12–15. (**d**,**e**) Time spent immobile in the tail suspension test and the forced swimming test, as an indication of depression-like phenotypes. *N* = 13–21. (**f**) Percent inhibition of acoustic startle at 74, 78, and 82 dB prepulse intensities, as an indication of sensory gating function. *N* = 12–15. Each point represents the mean  ±  SEM (*P < 0.05).

We also assessed some phenotypes related to human behavioral and psychiatric disease, such as hyperactivity, anxiety, depression, and schizophrenia-like phenotypes in *Chrna7* deficient mice. The locomotor activity in the open field assay was similar between *Chrna7* deficient and wildtype mice (shown in Fig. 3a, Two-WAY ANOVA on total distance, genotype effect *F* [2,75] = 1.95, *P* = 0.15). However, male mice ran a significantly longer distance than females (Two-WAY ANOVA, sex effect *F* [1,75] = 8.32, *P = 0.01). Interaction between genotype and sex was not significant (*F* [2,75] = 0.20, *P* = 0.82). The deficient mice were indistinguishable from wildtype in anxiety level, as tested in the elevated plus maze (Fig. 3c, Two-Way ANOVA on percent time in open arms, genotype effect *F* [2,75] = 0.85, *P* = 0.43; sex effect *F* [1,75] = 1.44, *P* = 0.23; genotype x sex interaction *F* [2,75] = 0.99, *P* = 0.38) and open field assay (Fig. 3b, Two-Way ANOVA on center/total distance, genotype effect *F* [2,75] = 0.77, *P* = 0.47; genotype x sex interaction *F* [2,75] = 0.50, *P* = 0.61). However, male mice ran a significantly longer distance in the center of the field than females (Two-WAY ANOVA, sex effect *F* [1,75] = 4.12, *P = 0.05). As seen in Fig. 3d and e, *Chrna7* null mice demonstrated no remarkable differences in duration of time being immobile, assessed in the tail suspension test and the forced swimming test (Two-Way ANOVA on time immobile in tail suspension test, genotype effect *F* [2,93] = 2.71, *P* = 0.07; sex effect *F* [1,93] = 0.02, *P* = 0.88; genotype x sex *F* [2,93] = 0.45, *P* = 0.64. Two-Way ANOVA on time immobile in forced swimming test, genotype effect *F* [2,93] = 3.54, *P = 0.03, followed by *post hoc* HET vs. KO, *P < 0.05; sex effect *F* [1,93] = 1.62, *P* = 0.21; genotype x sex *F* [2,93] = 1.19, *P* = 0.31). In addition, we found no sex or genotype effect on sensory gating (Fig. 3f, linear mixed mode followed by evaluation of significance by ANOVA, genotype effect *F* [2,75] = 0.42, *P* = 0.66; sex effect *F* [1,75] = 0.91, *P* = 0.34; genotype x sex interaction *F* [2,75] = 1.80, *P* = 0.17; prepulse intensity effect *F* [2,160] = 239.58, *P* < 0.0001). Previous reports on PPI that show inconsistent findings^17^,^21^,^23^ may be due to the small effect of *Chrna7* on prepulse inhibition, the different sex composition, and/or the small number of animals used in their study. Taken together, results from these assays suggest that *Chrna7* deficient mice have no phenotypic differences in hyperactivity, anxiety, depression-like behaviors, or sensory-gating.

**Figure 4:**
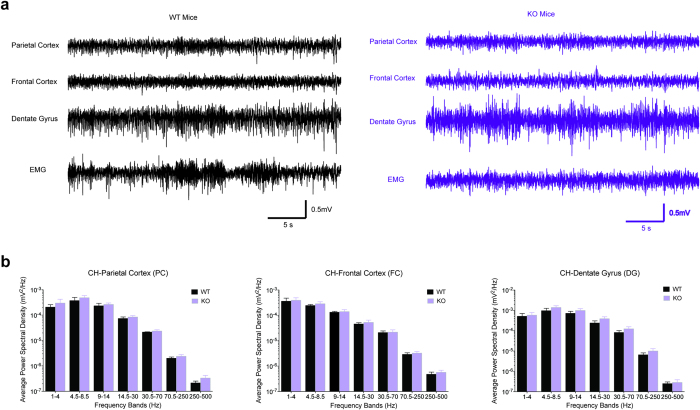
Normal EEG in *Chrna7* deficient mice. *N* = 5–7.(**a**) Representative EEG and EMG traces from a WT and a KO mouse. (**b**) Average power spectral density of EEG signals from the parietal cortex, frontal cortex, and dentate gyrus of WT and KO mice (*P* = 0.55, 0.73, and 0.41, respectively).

To characterize the brain activity of the *Chrna7* KO mice, we performed video-electroencephalography (EEG) and electromyography (EMG) recordings in freely-moving mice. We did not observe abnormal cortical discharges or electrographic seizures in KO mice. We further analyzed the power spectral density (PSD) of the EEG signals from the parietal cortex, frontal cortex, and hippocampal dentate gyrus. PSD within the frequency range of 0–500 Hz did not show a significant difference between WT and KO mice (Fig. 4), indicating that Chrna7 deficiency did not alter the EEG. Given that we only recorded EEGs from one wildtype and two *Chrna7* knockout male mice, our result is female-biased. However, data mining of previous literature suggests a lack of gender difference in patients with 15q13.3 microdeletion syndrome, with collective seizure occurrences of 30/79 and 33/63 in male and female patients respectively^12^,^13^,^36^,^37^,^38^,^39^,^40^,^41^,^42^,^43^,^44^,^45^,^46^,^47^,^48^,^49^,^50^,^51^,^52^,^53^,^54^,^55^,^56^.

## Discussion

*CHRNA7* has been associated with several neuropsychiatric and behavioral disorders, such as schizophrenia, bipolar disorder, autism, Alzheimer’s disease, Parkinson’s disease, and 15q13.3 microdeletion and duplication syndromes^5^,^6^,^7^,^8^,^9^,^10^,^11^,^12^,^13^,^14^,^15^. Previous studies showed that null mutations of *Chrna7* predominantly affect working memory and attention span in mice^19^,^20^,^21^. However, there was little investigation of the phenotypes related to human neuropsychiatric conditions and social behaviors in those mice. We assessed the effect of *Chrna7* deficiency in mice using a variety of behavioral tests relevant to autism spectrum disorder, aggression, depression, etc. The principal finding reported here is that loss of function in *Chrna7* is not sufficient to cause statistically significant social behavioral or neuropsychiatric-like alterations in mice. Furthermore, we looked beyond behavioral phenotypes, and did not find any evidence for electrophysiological phenotypical difference.

Compared to human phenotypes associated with 15q13.3 microdeletion syndrome, *Chrna7* null mice show fewer and subtler phenotypical differences, if they manifest any neurobehavioral abnormalities at all (Table 1). The reason for the discrepancy between human individuals and mouse models is unknown at this time. Several possibilities for this discrepancy include: 1) Possible compensation by other nAChR subunits, which may be more pronounced in mice than in humans. 2) Strain-related effects, with some genetic modifiers necessary for phenotypic expression of *Chrna7* deletion not being present in the C57BL/6 J mice. 3) Functions of *CHRNA7*, or the neural circuits affected by *CHRNA7* not being equivalent in mice and humans. For example, the human-specific gene *CHRFAM7A*, which consists of a partial duplication of *CHRNA7* and *FAM7A*, may play an important role in cognition and behavior. 4) Other genes in the genomic locus may account for the phenotypes associated with 15q13.3 microdeletion syndrome. Small deletion cases have been described with similar clinical manifestations, but these cases involved *CHRNA7* deletion, as well as the partial deletion of an immediately adjacent gene, *OTUD7A*. The consequences of *OTUD7A* loss of function have not been studied, and individuals with mutations of only *OTUD7A* have not been reported in the literature.

In summary, *Chrna7* knockout in mice does not recapitulate the spectrum of neurobehavioral phenotypes observed in human individuals with 15q13.3 microdeletion syndrome. Further work is required to investigate which other genes at the 15q13.3 chromosomal locus may contribute to disease phenotypes, or whether there are critical differences in the relevance of the alpha7 nicotinic receptor in humans compared with mice.

## Additional Information

**How to cite this article**: Yin, J. *et al. Chrna7* deficient mice manifest no consistent neuropsychiatric and behavioral phenotypes. *Sci. Rep.* **7**, 39941; doi: 10.1038/srep39941 (2017).

**Publisher's note:** Springer Nature remains neutral with regard to jurisdictional claims in published maps and institutional affiliations.

## Figures and Tables

**Table 1:** Phenotype comparison between 15q13.3 microdeletion patients and *Chrna7* deficient mice.

Phenotypes of 15q13.3 microdeletion patients	Prevalence in patients based on 79 males and 63 females*	Phenotypes of *Chrna7* deficient mice
Developmental delay or intellectual disability	87% in male, 83% in female	No phenotypic difference
Epilepsy/History of seizures	38% in male, 52% in female	No phenotypic difference
Mood disorder Anxiety Depression Aggression (predominantly affects males)	21% in male, 14% in female	No phenotypic difference
Hyperactivity or attention deficit	22% in male, 10% in female	Attention deficit, only male mice tested**
Autism	23% in male, 3% in female	No gross phenotypic difference***
Schizophrenia	Unknown	No phenotypic difference

*Data obtained based on the following references^12^,^13^,^36^,^37^,^38^,^40^,^41^,^42^,^44^,^45^,^46^,^47^,^49^,^50^,^51^,^52^,^53^,^54^,^55^,^56^,^57^,^58^,^59^. **Based on^18^,^20^,^21^,^60^. ***The only significant difference seen is genotype by sex effect in the marble burying test (this study).
